# Reduced platelet mitochondrial respiration and oxidative phosphorylation in patients with post COVID-19 syndrome are regenerated after spa rehabilitation and targeted ubiquinol therapy

**DOI:** 10.3389/fmolb.2022.1016352

**Published:** 2022-10-21

**Authors:** Zuzana Sumbalová, Jarmila Kucharská, Zuzana Rausová, Patrik Palacka, Eleonóra Kovalčíková, Timea Takácsová, Viliam Mojto, Plácido Navas, Guillermo Lopéz-Lluch, Anna Gvozdjáková

**Affiliations:** ^1^ Comenius University in Bratislava, Faculty of Medicine, Pharmacobiochemical Laboratory of 3rd Department of Internal Medicine, Bratislava, Slovakia; ^2^ Comenius University in Bratislava, Faculty of Medicine, 2nd Department of Oncology, Bratislava, Slovakia; ^3^ Sanatorium of Dr Guhr, Tatranská Polianka, Slovakia; ^4^ Comenius University in Bratislava, Faculty of Medicine, 3rd Department of Internal Medicine, Bratislava, Slovakia; ^5^ Centro Andaluz de Biología del Desarrollo, Universidad Pablo de Olavide-CSIC-JA and CIBERER, Instituto de Salud Carlos III, Sevilla, Spain

**Keywords:** platelets, mitochondrial oxidative phosphorylation, spa rehablitation, ubiquinol, pulmonary function, post COVID-19 syndrome

## Abstract

European Association of Spa Rehabilitation recommend spa rehabilitation for patients with post COVID-19 syndrome (post C-19). We studied effects of special mountain spa rehabilitation program and its combination with ubiquinol (reduced form of coenzyme Q_10_—CoQ_10_) supplementation on pulmonary function, clinical symptoms, endogenous CoQ_10_ levels, and platelet mitochondrial bioenergetics of patients with post C-19. 36 patients with post C-19 enrolled for rehabilitation in mountain spa resort and 15 healthy volunteers representing the control group were included in this study. 14 patients with post C-19 (MR group) were on mountain spa rehabilitation lasting 16–18 days, 22 patients (MRQ group) were supplemented with ubiquinol (2 × 100 mg/day) during the rehabilitation and additional 12–14 days at home. Clinical symptoms and functional capacity of the lungs were determined in the patients before and after the spa rehabilitation program. Platelet bioenergetics by high-resolution respirometry, plasma TBARS concentration, and CoQ_10_ concentration in blood, plasma and platelets were evaluated before and after the spa rehabilitation program, and in 8 patients of MRQ group also after additional 12–14 days of CoQ_10_ supplementation. Pulmonary function and clinical symptoms improved after the rehabilitation program in both groups, 51.8% of symptoms disappeared in the MR group and 62.8% in the MRQ group. Platelet mitochondrial Complex I (CI)-linked oxidative phosphorylation (OXPHOS) and electron transfer (ET) capacity were markedly reduced in both groups of patients. After the rehabilitation program the improvement of these parameters was significant in the MRQ group and moderate in the MR group. CI-linked OXPHOS and ET capacity increased further after additional 12–14 days of CoQ_10_ supplementation. CoQ_10_ concentration in platelets, blood and plasma markedly raised after the spa rehabilitation with ubiquinol supplementation, not in non-supplemented group. In the MRQ group all parameters of platelet mitochondrial respiration correlated with CoQ_10_ concentration in platelets, and the increase in CI-linked OXPHOS and ET capacity correlated with the increase of CoQ_10_ concentration in platelets. Our data show a significant role of supplemented ubiquinol in accelerating the recovery of mitochondrial health in patients with post C-19. Mountain spa rehabilitation with coenzyme Q_10_ supplementation could be recommended to patients with post C-19. This study was registered as a clinical trial: ClinicalTrials.gov ID: NCT05178225.

## 1 Introduction

Mountain spa rehabilitation is beneficial for pulmonary diseases, for improving fatigue, joint pain, psychological stress, depression, sleep disorders, and quality of life of patients with various diseases, mostly with chronic lung disease (Gvozdjáková et al., 2021).

In March 2020 the World Health Organization declared a global pandemic caused by the SARS-CoV-2 virus. The main symptoms after acute phase of COVID-19 include shortness of breath, general fatigue, exhaustion, headaches, muscle and joint pain, cough, hair, taste and smell loss, sleep and memory disturbances, depression, sensitivity to sound and light. If the symptoms persist for several months after acute COVID-19, these health complications are known as post COVID-19 syndrome (post C-19). Higher incidence of post C-19 is in aged subjects and in patients with comorbidities such as diabetes mellitus, obesity, cardiovascular disease, chronic lung disease, cancer (Lopez-Lluch 2017; Fernandez-Ayala et al., 2020; Huang et al., 2020; Li et al., 2020; Zhang and Liu, 2020; Ganji and Reddy 2021). Respiratory symptoms include dyspnea and reduced blood oxygen saturation. Post-hospitalization pulmonary spa rehabilitation should be considered in all patients with post C-19 (Siddiq et al., 2020; Wang et al., 2020; Maccarone and Mesiero 2021).

Platelets are accessible source of mitochondria and their bioenergetics may reflect systemic mitochondrial health (Kramer et al., 2014; Nguyen et al., 2019; Hoppel et al., 2021). Our recent study showed a deficit of mitochondrial bioenergetic function and a reduced concentration of endogenous coenzyme Q_10_ (CoQ_10_) in platelets of non-hospitalized, non-vaccinated patients 3–6 weeks after acute COVID-19 (Sumbalová et al., 2022).

In this project we studied effects of mountain spa rehabilitation program and its combination with ubiquinol (reduced form of CoQ_10_) supplementation on pulmonary function, clinical and psychological symptoms, endogenous CoQ_10_ levels, and platelet mitochondrial bioenergetics of patients with post C-19. We tested the hypothesis that ubiquinol with mountain spa rehabilitation can accelerate the recovery of patients with post C-19 (Gvozdjáková et al., 2020; Gvozdjáková et al., 2021).

## 2 Materials and methods

### 2.1 Study groups

#### 2.1.1 The control group

Consisted of 15 healthy individuals (6 men and 9 women), aged from 38 to 67 years with a mean age of 51.3 ± 2.3 years, without history of COVID-19. Exclusion criteria were obesity, smoking, regular alcohol consumption. Recent CoQ_10_ supplementation was an exclusion criterion for all study groups.

#### 2.1.2 The patients with post COVID-19 syndrome (post C-19)

From all over Slovakia enrolled for rehabilitation in Sanatorium of Dr. Guhr in Tatranská Polianka, High Tatras, Slovakia in May 2021 were randomly divided into two groups. 1)The **MR** (mountain spa rehabilitation) group consisting from 14 patients (8 men and 6 women) with the mean age 51.3 ± 2.3 years was subjected to a spa rehabilitation program for 16–18 days. 2)The **MRQ** group consisting from 22 patients (14 men and 8 women) with the mean age 57.8 ± 2.5 years was during the 16–18 days of the spa rehabilitation program supplemented with CoQ_10_ (ubiquinol) 2 × 100 mg/day, and the supplementation with CoQ_10_ continued for another 12–14 days after leaving the mountain spa resort. CoQ_10_ (ubiquinol) in capsules was taken by patients at breakfast and lunch during or after the meal. Two patients from MRQ group had to leave mountain spa due to unexpected health complications and therefore only 20 patients are in the MRQ2 group.

The total number of patients in the study was limited by the number of patients admitted and treated in the Sanatorium during the period reserved for this study. The patients included in the study were 3–7 months after hospitalization for a severe course of COVID-19 mostly with bilateral pneumonia and the need for oxygen therapy. The patients were with many persistent clinical symptoms (listed in Table 2), unable to work. During their stay in the Sanatorium, the patients used drugs for the treatment of lung, immunological, cardiovascular, nephrological, hepatological, neurological diseases, dyslipidemia and diabetes mellitus. Broncholytics were used by 13 patients, corticoids by 8 patients, anticoagulants by 14 patients, antihypertensives by 23 patients, hypolipidemic drugs by 7 patients (6 of them used statins). Antihistamines were used by 6 patients, and neurological drugs by 6 patients. Drug use was in similar proportions in both groups.

### 2.2 Comprehensive respiratory physiotherapy program in the Sanatorium

Patients with post C-19 completed a comprehensive respiratory physiotherapy program in the Sanatorium. After the initial medical examination, each patient was individually assigned to a group according to the risk and possibility of burdening the patient from a cardiopulmonary point of view, taking into account associated diseases. Each patient received individual respiratory physiotherapy once a week (30 min) aimed at adjusting the breathing pattern and therapy for specific patient problems, e.g. education on the use of a breathing aid for exhalation training or inspiratory muscles or cough management techniques. Furthermore, each patient completed 3–4 times a week group respiratory physiotherapy (30 min) aimed at learning the breathing pattern in various positions according to the indoor and outdoor program with gradual adaptation to the load under the supervision of a trained physiotherapist in a group of 5–10 participants formed according to risk. Group exercises were aimed at increasing cardiopulmonary capacity in the sense of aerobic exercise in the load zone of 40–60% VO_2_ max. The patients were taught to perceive the load (2–4 degree of the Borg scale) without going into exhaustion, without hyposaturation after the load or respiratory decompensation. The patients also completed strength exercises 1–2 times a week (30 min) aimed at muscle strength and special exercises for the deep stabilization system. In addition to the active respiratory program, the patients completed other rehabilitation procedures in terms of inhalation, electrotherapy, thermotherapy, mechanotherapy and hydrotherapy according to the patient’s state of health. 13 patients used oxygen therapy 3 times a day for 30 min, with O_2_ flow 3.5–4.5 L/min. During the stay in the Sanatorium, the patients had standardized diets according to their diagnosis (rational diet, diabetic diet, gluten-free diet, etc.). The standard length 16–18 days of the hospitalization in the Sanatorium is sufficient for learning and acquiring new habits of movement therapy, which the patient should continue at home according to the instructions.

### 2.3 Study design

Blood samples from the patients of both groups were taken the first morning after the arrival to the Sanatorium (MR1, MRQ1) and the last morning before departure (MR2, MRQ2). At the same time points lungs function was evaluated by 6-min walking test (6MWT), exercise dyspnea and blood oxygen saturation (SpO_2_), and the patients filled out the questioannaire on their current clinical symptoms. From the blood samples blood counts, blood glucose, and blood lipid parameters were determined, and platelets (PLT) were isolated for determination of mitochondrial respiration. Aliquots of blood, plasma and PLT samples were frozen for determination of CoQ_10_ content, and plasma samples were frozen for evaluation of TBARS.

In 8 patients from MRQ group, the mitochondrial respiration in PLT, TBARS and CoQ_10_ concentration in blood, plasma and PLT were determined also after additional 12–14 days of CoQ_10_ supplementation at home after completing the mountain spa rehabilitation program (MRQ3).

### 2.4 Functional capacity of the lungs

The functional capacity of the lungs was evaluated on the basis of 6-min walking test (6MWT), exercise dyspnea in 6MWT evaluated by Borg scale (BS) points, and blood oxygen saturation (SpO_2_) before and after 6MWT. The results are summarized in Figure 2 and Table 1.

**TABLE 1:** Effect of rehabilitation program on blood oxygen saturation of patients with post C-19.

Parameter	MR1	MR2	MRQ1	MRQ2
	(*N* = 10)	(*N* = 10)	(*N* = 14)	(*N* = 14)
SpO_2_ (%)				
Before 6MWT	94.1 ± 0.59	94.1 ± 0.72	93.2 ± 0.67	94.2 ± 0.52
After 6MWT	94.9 ± 0.60	93.9 ± 0.78	90.5 ± 1.29	92.9 ± 0.59

MR1, MRQ1—the group of patients with post C-19 at the beginning of the study; MR2, MRQ2—the groups of patients with post C-19 after 16–18 days of rehabilitation program without/with CoQ_10_ supplementation.

### 2.5 Clinical symptoms

Clinical symptoms were recorded by patient questionnaire filled out before and after the rehabilitation program without/with CoQ_10_ supplementation. The clinical symptoms included dry cough, difficulty breathing, shortness of breath in rest, chills, heart palpitation, weakness, overall fatigue, chest, back, muscle and joint pain, headache, the loss of taste and smell, weight loss, hearing impairment, visual disturbance, psychical disturbances and depression. The results are summarized in Table 2.

**TABLE 2:** Effect of rehabilitation program on clinical symptoms of 10 patients with post C-19 from MR group and 20 patients from MRQ group.

Clinical symptom	MR1	MR2	Improved	MRQ1	MRQ2	Improved
	*N* of symptoms	*N* of symptoms	%	*N* of symptoms	*N* of symptoms	%
Dry cough	4	3	*25*	9	2	*78*
Shortness of breath	6	6	*0*	13	6	*54*
Difficulty breathing	6	3	*50*	13	8	*38*
Elevated temperature	2	0	*100*			
Chills	2	1	*50*	3	0	*100*
Heart palpitation	3	1	*67*	10	4	*60*
Respiratory support with O_2_				3	0	*100*
Weakness				2	1	*50*
Overall fatigue	7	2	*72*	17	7	*59*
Malaise	2	2	*0*	1	0	*100*
GIT problems				1	1	*0*
Diarrhea	1	1	*0*	3	1	*67*
Chest pain	3	1	*67*	9	3	*67*
Muscle and joint pain	10	5	*50*	11	4	*64*
Back pain				7	3	*57*
Headache	4	0	*100*	8	2	*75*
Loss of taste and smell				1	1	*0*
Weight loss	1	1	*0*	4	0	*100*
Hearing impairment	2	0	*100*	4	0	*100*
Visual disturbance	3	1	*66*	6	5	*17*
Depression, anxiety				3	0	*100*
Memory problems				1	0	*100*
**Total *N* of symptoms**	**56**	**27**		**129**	**48**	
**%**	100%	48.2%	**51.8%**	100%	37.2%	**62.8%**

MR1, MRQ1—the group of patients with post C-19 at the beginning of the study; MR2, MRQ2—the groups of patients with post C-19 after 16–18 days of rehabilitation program without/with CoQ_10_ supplementation. The % calculation was as follows: improved % was calculated for each symptom separately; the total *N* of symptoms in the groups at the beginning of the study (MR1, MRQ1) served as 100% for the calculation of symptoms % persisting after rehabilitation program without/with CoQ_10_ supplementation. The improvement was calculated from the difference of 100% and persisting % of symptoms in each group.

Bold values highlight total number of symptoms and total proportions of improvement.

### 2.6 Blood count and biochemical parameters

In all control subjects and patients with post COVID-19 syndrome before and after rehabilitation program without/with CoQ_10_ supplementation blood counts, blood glucose, and blood lipid parameters were determined at clinical laboratory. The results are summarized in Table 3.

**TABLE 3:** Blood count and biochemical parameters in control subjects and patients with post C-19, the effect of rehabilitation program without/with ubiquinol supplementation.

Parameter	Control	MR1	MR2	MR1 vs. C	MR2 vs. MR1	MRQ1	MRQ2	MRQ1 vs. C	MRQ2 vs. MRQ1
				*p*-value	*p*-value			*p*-value	*p*-value
**GLU** (mmol/L)	5.13 ± 0.17	6.17 ± 0.60	5.35 ± 0.31	ns	0.069	5.64 ± 0.21	6.01 ± 0.33	ns	ns
**Blood count**									
**WBC** (10^9^/L)	6.23 ± 0.48	6.99 ± 0.72	6.59 ± 0.64	ns	ns	8.12 ± 0.31	8.03 ± 0.36	**0.026**	ns
**NE** (10^9^/L)	3.34 ± 0.35	3.01 ± 0.28	2.73 ± 0.19	ns	ns	4.44 ± 0.23	4.13 ± 0.29	0.074	ns
**LY** (10^9^/L)	2.11 ± 0.17	2.68 ± 0.18	2.59 ± 0.17	0.052	ns	3.01 ± 0.18	2.99 ± 0.26	**0.048**	ns
**MO** (10^9^/L)	0.522 ± 0.041	0.519 ± 0.048	0.513 ± 0.053	ns	ns	0.542 ± 0.044	0.603 ± 0.037	ns	0.088
**RBC** (10^9^/L)	4.66 ± 0.12	4.62 ± 0.12	4.80 ± 0.09	ns	**0.008**	4.64 ± 0.12	4.91 ± 0.14	ns	**0.00012**
**HCT** (ratio)	0.411 ± 0.009	0.418 ± 0.01	0.438 ± 0.008	ns	**0.003**	0.423 ± 0.009	0.459 ± 0.014	ns	**0.000002**
**PLT** (10^9^/L)	247.5 ± 16.7	213.9 ± 14.9	219.1 ± 11.2	ns	ns	226.2 ± 11.3	231.1 ± 12.3	ns	0.092
**MCV** (fL)	87.14 ± 0.67	90.31 ± 1.26	91.21 ± 1.26	**0.048**	**0.009**	91.30 ± 0.98	92.96 ± 1.31	0.068	**0.00001**
**MCH** (pg)	29.95 ± 0.29	31.58 ± 0.49	31.10 ± 0.41	**0.028**	0.079	31.57 ± 0.34	31.59 ± 0.46	**0.046**	ns
**MCHC** (g/L)	343.7 ± 2.6	349.6 ± 2.0	341.08 ± 1.21	ns	**0.002**	344.3 ± 1.6	339.8 ± 0.8	ns	**0.020**
**HGB** (g/L)	140.7 ± 3.4	145.5 ± 3.4	149.23 ± 2.89	ns	0.056	146.3 ± 3.1	155.4 ± 4.8	ns	**0.00009**
**CHOL** (mmol/L)	5.32 ± 0.27	5.51 ± 0.30	5.76 ± 0.397	ns	ns	5.31 ± 0.22	5.26 ± 0.25	ns	ns
**HDL-CH** (mmol/L)	1.41 ± 0.13	1.10 ± 0.09	1.121 ± 0.099	0.062	ns	1.20 ± 0.07	1.11 ± 0.06	ns	ns
**LDL-CH** (mmol/L)	3.09 ± 0.26	3.37 ± 0.29	3.344 ± 0.316	ns	ns	3.31 ± 0.18	3.16 ± 0.21	ns	ns
**TAG** (mmol/L)	2.05 ± 0.51	2.49 ± 0.56	3.224 ± 0.954	0.11	ns	2.05 ± 0.16	2.30 ± 0.31	ns	ns

Legends: GLU, glucose; WBC, white blood cells; NE, neutrophils; LY, lymphocytes; MO, monocytes; RBC, red blood cells; HCT, hematocrit, MVC- mean corpuscular volume; MCH, mean corpuscular hemoglobin; MCHC, mean corpuscular hemoglobin concentration; HGB, hemoglobin; CHOL, total cholesterol, HDL-CH—HDL, cholesterol, LDL-CH—LDL, cholesterol; TAG—triacylglycerols. Control—the control group; MR1, MRQ1—the groups of patients with post C-19 at the beginning of the study; MR2, MRQ2—the groups of patients with post C-19 after 16–18 days of rehabilitation program without/with CoQ_10_ supplementation.; The differences between MR1, MQ1 and the control group, and between MR1 and MR2, and MRQ2 and MRQ1 groups are statistically evaluated.

Bold values highlight statistically significant differences.

### 2.7 Platelets preparation

Platelets were isolated from whole blood (Sumbalova et al., 2016) as described previously (Gvozdjáková et al., 2019). Blood samples collected by venipuncture into two 9 mL K3EDTA (tripotassium ethylenediaminetetraacetic acid) tubes were transported at 25°C room temperature to the laboratory and centrifuged at room temperature at 200×*g* for 10 min using swing-out rotor, with the brakes off. Platelet-rich plasma was transferred into a new plastic tube and mixed with 100 mM EGTA (ethylene glycol-bis (2-aminoethylether)-N,N,N′,N′-tetraacetic acid) to a final concentration 10 mmol/L. The pellet after centrifugation at 1,200×*g* containing PLT, was washed with 4 mL of DPBS (Dulbecco’s Phosphate-Buffered saline) plus 10 mM EGTA and resuspended in 0.4 mL of the same solution. Immediately after isolation, the PLT subsamples (10 times diluted) were transported for counting in a thermo-insulating container at room temperature by car to the National Institute for Pediatric Respiratory Diseases and Tuberculosis, n.o., Dolný Smokovec which was 7 km away from the Sanatorium. The PLT suspension was counted on hematological analyzer Mindray BC-3600 (Mindray, China) (Sumbalova et al., 2022). The exact PLT count in the isolated cell suspension allowed measurements with a standard concentration of PLT. Aliquots of PLT suspension were frozen for later evaluation of citrate synthase activity, which was used for normalization of the respiration. A maximum of four to six blood samples from patients could be processed each day (Monday to Friday), to ensure that respiration in freshly isolated PLT was measured within 2 h of isolation. All procedures were performed in accordance to currently published research (Sjövall et al., 2013; Sumbalova et al., 2016).

### 2.8 High-resolution respirometry

**FIGURE 1:**
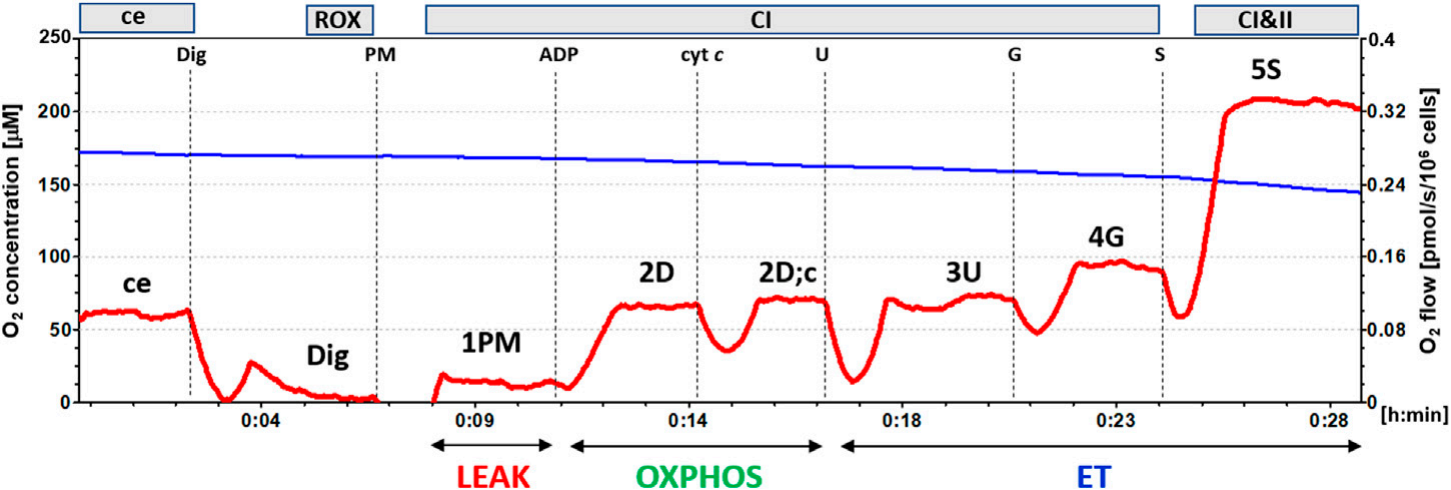
The trace from the measurement of platelet mitochondrial respiration following SUIT reference protocol 1 (Doerrier et al., 2018). The blue line represents oxygen concentration (µM), and the red trace oxygen consumption as flow per cells (pmol O_2_/s/10^6^ cells). The protocol includes following titration steps: ce—cells; Dig—Digitonin; PM—pyruvate + malate; ADP; cyt c—cytochrome c; U—the uncoupler FCCP; G—glutamate; S—succinate. All substrates were titrated in kinetically saturating concentrations, uncoupler FCCP was titrated in optimum concentration for evaluation of maximum O_2_ flow at given titration step. The evaluated respiratory capacities are marked according to the titration steps in the reference protocol 1 and correspond to following respiratory states: ce—routine respiration of intact cells; Dig—residual oxygen consumption (ROX) after permeabilization with digitonin; 1PM—LEAK respiration with CI-linked substrates pyruvate + malate; 2D—CI-linked OXPHOS capacity; 2D;c—CI-linked OXPHOS capacity after addition of cytochrome c; 3U—CI-linked electron transfer (ET) capacity with pyruvate + malate; 4G—CI-linked ET capacity with pyruvate + malate + glutamate; 5S—CI&II-linked ET capacity. (Author ZS, published in Gvozdjáková et al., 2022).

Mitochondrial function in isolated platelets was determined with the use of high-resolution respirometry (Pesta and Gnaiger 2012; Sjövall et al., 2013). The respiration was measured at 37°C in mitochondrial respiration medium MiR05 (Pesta and Gnaiger 2012) with addition of 20 mM creatine under continuous stirring at 750 rpm. For evaluation of platelet mitochondrial function, 250×10^6^ platelets were used in a 2 mL chamber of an O2k-Respirometer (Oroboros Instruments, Austria) and substrate-uncoupler-inhibitor titration (SUIT) reference protocol 1 (Doerrier et al., 2018) was applied. The trace from a typical measurement is in Figure 1.

In intact platelets *Routine* respiration was measured (ce). After permeabilization of cell membrane with digitonin (0.20 µg/10^6^ cells) residual oxygen consumption (ROX) was determined. After subsequent addition of CI-linked substrates pyruvate (5 mM) and malate (2 mM), mitochondrial CI-linked LEAK respiration was measured (1PM). After addition of saturating ADP concentration (1 mM), CI-linked OXPHOS capacity was determined (2D). Addition of cytochrome *c* (cyt *c*) (10 μM) was used as a test for the integrity of outer mitochondrial membrane (2D; c). Addition of uncoupler FCCP at optimum concentration (0.5 μM) allowed determination of maximum oxidative capacity with CI-linked substrates, CI-linked electron transfer (ET) capacity (3U). Subsequent addition of 10 mM glutamate completed CI-linked pathway (4G), adding a small share of CII-linked pathway to the O_2_ flux (Sumbalova et al., 2014). After addition of CII-linked substrate succinate (S, 10 mM) noncoupled CI&II-linked ET capacity was determined (5S). For evaluation of mitochondrial respiration, the respiratory rate after digitonin, representing residual oxygen consumption (ROX) was subtracted from all respiratory rates.

### 2.9 Citrate synthase activity

The activity of citrate synthase as a mitochondrial marker was evaluated by spectrophotometric method (Srere 1969; Eigentler at al., 2020). All assays were conducted with an UV-Visible spectrophotometer Biochrom 4060 using 0.3 mL sample cuvettes at 25°C. The reaction mixture contained 100 mM Tris-HCl pH = 8.1, 101 mM 5,5′-dithiobis (2-nitrobenzoic acid), 0.25% Triton X-100, and 0.3 mM acetyl-CoA. After addition of platelets from thawed suspension (15x10^6^ cells/measurement), the reaction was initiated by the addition of 0.5 mM oxaloacetate and followed at 412 nm for 1.5 min. The activity of CS (in µmol/min/10^6^ cells = IU) was calculated from the rate of concentration change using extinction coefficient of thionitrobenzoic acid 13.6/mM/cm. All parameters of oxygen consumption were evaluated as *J*O_2_/CS (pmol/s/IU).

### 2.10 Coenzyme Q_10_

CoQ_10-TOTAL_ (ubiquinol + ubiquinone) in whole blood, plasma and isolated platelets were determined using HPLC method with UV detection (Lang et al., 1986), modified by authors (Kucharská et al., 1998; Mosca et al., 2002). For the oxidation of ubiquinol to ubiquinone, 100 µL of 1,4-benzoquinone (2 mg/mL double distilled water—daily fresh) was added to 500 µL of blood or plasma sample and vortexed for 10 s. After 10 min of incubation at room temperature 2 mL of the hexane/ethanol (5/2, v/v) mixture was added. The sample was shaken for 5 min and centrifuged at 1,000 *g* for 5 min. The hexane layer was separated and the extraction procedure was repeated with 1 mL of the extraction mixture. Collected organic layers were evaporated under nitrogen at 50°C. The residues were taken up in 99.9% ethanol and injected into the reverse phase of HPLC column (SGX C18, 7 μm, Tessek Ltd.). Elution was per-formed with methanol/acetonitrile/ethanol (6/2/2, v/v/v) at a flow rate 0.9 mL/min. The concentrations of CoQ_10-TOTAL_ were detected with an UV-detector at 275 nm, using an external standard (Sigma). Data were collected and processed with a CSW32 chromatographic station (DataApex Ltd.). The concentrations of CoQ_10-TOTAL_ were calculated in µmol/L.

The isolated platelets (150–250x10^6^ cells) were disintegrated with 500 µL of cold methanol (Niklowitz et al., 2004). Oxidation of ubiquinol to ubiquinone was performed with 1,4 benzoquinone as described for plasma samples. The cell supension was extracted with 2 mL of hexane by shaking for 5 min. After centrifugation, the organic layer was separated, evaporated and processed as desribed above. Concentrations of CoQ_10-TOTAL_ were calculated in pmol/10^9^ cells.

### 2.11 TBARS

Thiobarbituric acid reactive substances (TBARS) as a parameter of oxidative stress was determined by the spectrophotometric method (Janero and Bughardt 1989). Plasma samples were mixed with ice-cold 76% trichloroacetic acid (TCA) and 1.07% thiobarbituric acid. Samples were incubated at 100°C for 30 min and after cooling down, 90% TCA was added. After centrifugation at 2,200 *g* for 15 min, the absorbance of supernatant was measured at 532 nm with an UV-Visible spectrophotometer Biochrom 4060, and the concentration in μmol/L was calculated.

### 2.12 Data analysis

The results in tables and bar graphs are expressed as mean ± standard error of mean (sem). The graphs with correlations show individual data points. The data were statistically evaluated with GraphPad Prism 6 for Windows. Normality was tested with the D'Agostino & Pearson omnibus normality test. The vast majority of parameters followed normal distribution allowing the use of parametric tests. Unpaired Student´s *t*-tests were applied to evaluate the difference between the parameters of the MR1, the MRQ1 and the control group. Bonferroni correction was applied to account for multiple testing. Paired Student´s t-tests were used for evaluation the difference between the MR1 and the MR2, the MRQ1 and the MRQ2, and between the MRQ1 and the MRQ3 groups. In case the variables did not follow normal distribution, the corresponding non-parametric tests were used: Mann Whitney test instead of unpaired *t*-test, and Wilcoxon matched-pairs signed rank test instead of paired *t*-test. *p*-values < 0.05 were considered statistically significant.

## 3 Results

### 3.1 Effect of mountain spa rehabilitation program without/with ubiquinol supplementation on lungs function of patients with post C-19

**FIGURE 2:**
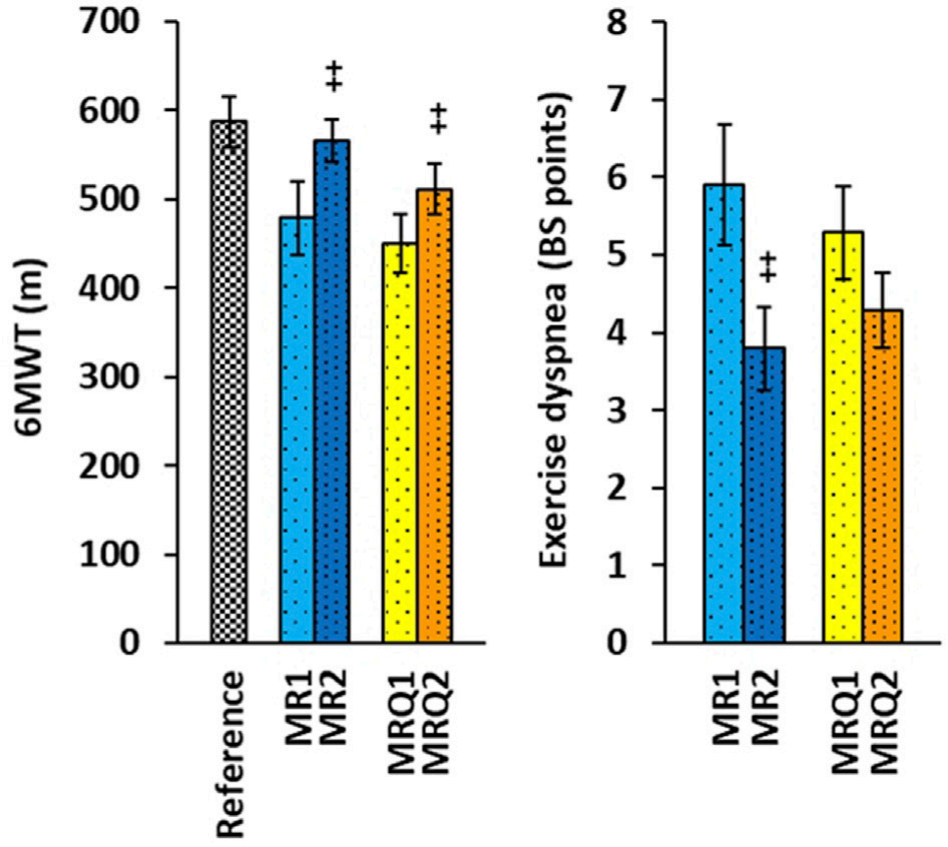
The effect of rehabilitation program on 6MWT and exercise dyspnea of patients with post C-19. Reference—the reference values for healthy subjects of age 50–59 years (Casanova et al., 2011). MR1, MRQ1—the group of patients with post C-19 at the beginning of the study; MR2, MRQ2—the groups of patients with post C-19 after 16–18 days of rehabilitation program without/with CoQ_10_ supplementation. +*p* < 0.05, ++*p* < 0.01 vs. the same group at the beginninng of the study.

The functional capacity of the lungs was evaluated by three parameters: 6-min walking test (6MWT), Borg scale (BS) for exercise dyspnea, and blood oxygen saturation (SpO_2_) before and after 6MWT. Only 10 patients from MR group and 14 patients from MRQ group were included in these tests before and after completion of the rehabilitation program without/with CoQ_10_ supplementation. The results of these functional tests are summarized in Figure 2 and Table 1. The distance walked in 6MWT increased by 87.2 ± 30.1 m in the MR2 group vs*.* the MR1 group (*p* = 0.004) and by 61.4 ± 18.1 m in the MRQ2 group vs*.* the MRQ1 group (*p* = 0.003). Exercise dyspnea measured by BS decreased by 2.1 ± 0.55 points in the MR2 vs*.* the MR1 group (*p* = 0.004) and by 1.0 ± 0.48 point in the MRQ2 vs*.* the MRQ1 group (*p* = 0.08). Normal blood oxygen saturation (SpO_2_) is between 95—100%. The values between 92% and 88% are considered safe and normal for patients with moderate to severe chronic obstructive pulmonary disease. The values of blood oxygen saturation in the patients from both the MR and the MRQ group were between 88 and 98%. There were no significant changes in oxygen saturation (SpO_2_) after the rehabilitation program in either group (Table 1).

### 3.2 Effect of mountain spa rehabilitation program without/with ubiquinol supplementation on clinical symptoms of patients with post C-19

Ten patients from the MR group and twenty patients from the MRQ group filled out the questionnaire before and after the rehabilitation program, the results are summarized in Table 2.

The most abundant symptoms at the beginning of the study were overall fatigue, and muscle and joint pain. These symptoms significantly improved after rehabilitation program in both groups (Table 2). Difficulty breating, heart palpitation, chest pain and headache reported by patients at the beginning of the study improved markedly by similar ratio in both groups. Hearing impariment was completely resolved in patients of both groups. Dry cough and shortness of breath improved significantly in the MRQ group, not in the the MR group. Three patients from the MRQ group who initially required oxygen support did not need it after the rehabilitation program with CoQ_10_ supplementation (Table 2). Overall, after completing the rehabilitation program 51.8% symptoms disappeared in the MR group and 62.8% in the MRQ group.

### 3.3 Effect of mountain spa rehabilitation program without/with ubiquinol supplementation on blood count and biochemical parameters of patients with post C-19

The patients with post C-19 in the MRQ1 group had more white blood cells (WBC) compared to the control group (Table 3). Patients from both the MR1 and the MRQ1 groups had bigger erythrocytes (higher mean corpuscular volume, MCV) with higher content of hemoglobin (mean corpuscular hemoglobin, MCH) in comparison with controls. There was no significant difference in blood glucose or blood lipid parameters between the patients groups and the control group.

After mountain spa rehabilitation program the number of red blood cells (RBC), their size (MCV), hematocrit (HCT), and blood hemoglobin concentration (HGB) increased in both groups of patients reflecting physiological adaptation to moderate altitude. The increase in MCV, HCT and HGB was significantly higher in the MRQ group vs*.* the MR group (ΔMCV: 1.956 ± 0.293 vs*.* 0.892 ± 0.286 fL, *p* = 0.016; ΔHCT: 0.035 ± 0.005 vs*.* 0.020 ± 0.005, *p* = 0.044; ΔHGB: 9.06 ± 1.71 vs*.* 3.77 ± 1.78 g/L, *p* = 0.043). The rehabilitation program did not influence blood glucose or lipid parameters in either group (Table 3).

### 3.4 Effect of mountain spa rehabilitation program without/with ubiquinol supplementation on platelet mitochondrial function in patients with post C-19

**FIGURE 3:**
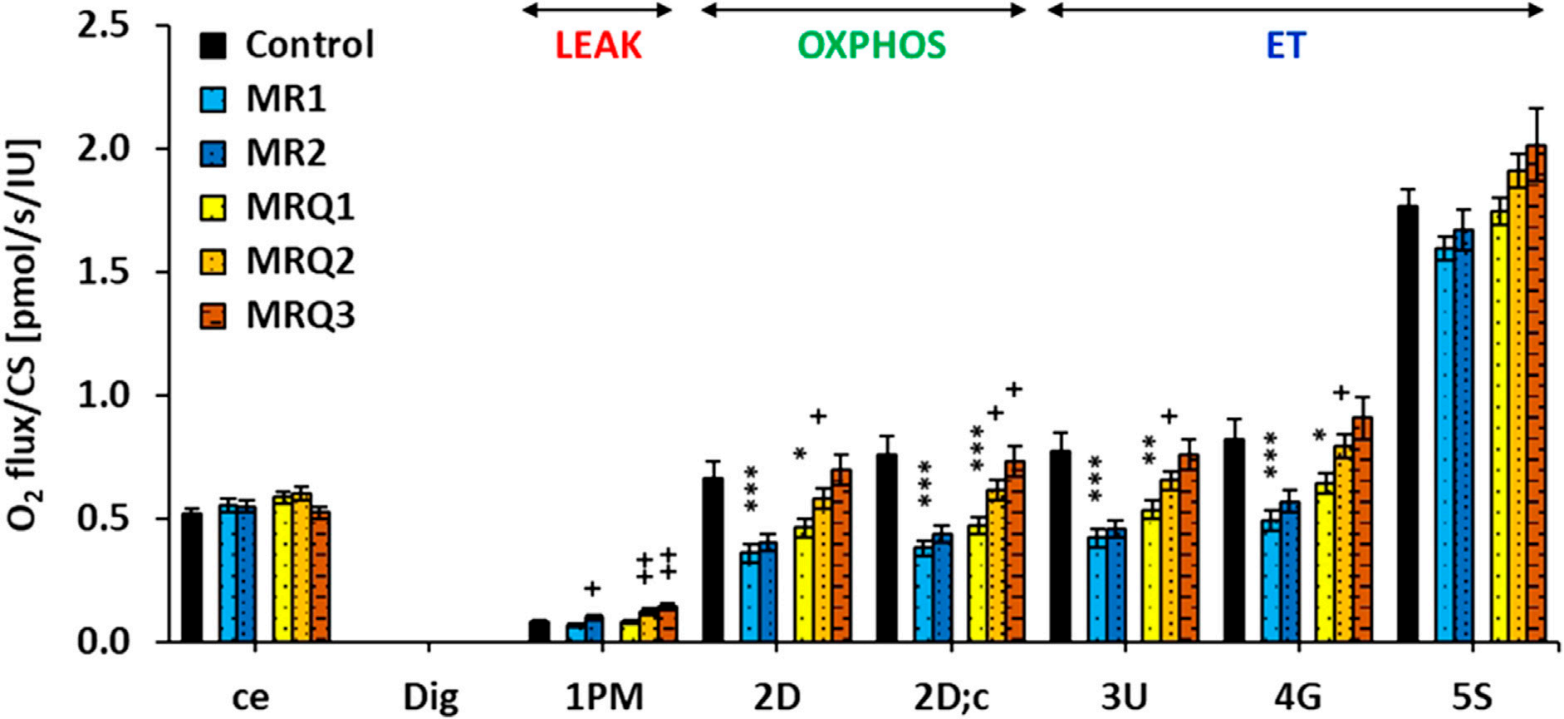
Platelet mitochondrial respiration in control subjects and patients with post C-19. The respiration was measured in freshly isolated platelets following substrate-uncoupler-inhibitor titration (SUIT) reference protocol 1 (Doerrier et al., 2018) and valuated as O_2_ flux per mitochondrial marker citrate synthase activity, *J*O_2_/CS (pmol/s/IU). A) The graph show mean ± sem of the respiratory capacities after titration step indicated on the *x*-axis: ce—intact cells; Dig—digitonin; 1p.m.—pyruvate plus malate; 2D—ADP—adenosine diphosphate; 2D; c—cytochrome c; 3U—uncoupler FCCP; 4G—glutamate; 5S—succinate. For linking the respiratory rates to respiratory states refer to Figure 1. Control—the control group; MR1, MRQ1 the groups of patients at the beginning of the study; MR2, MRQ2—the MR and MRQ group after 16–18 days of rehabilitation program without/with CoQ_10_ supplementation; MRQ3—the MRQ group after 12–14 days of completion of the rehabilitation program and continuation of CoQ_10_ supplementation at home. *p < 0.05, **p < 0.01, ***p < 0.01 vs*.* Control. +*p* < 0.05, ++*p* < 0.01 vs*.* the same group at the biginning of the study.

The results from platelet mitochondrial bioenergetics normalized per mitochondrial marker CS activity are shown in Figure 3.

Routine respiration of intact platelets *(ce)* was similar in all groups. In the MRQ3 group this parameter decreased to 90% of MRQ1 values (ns) (Figure 3). The respiration in non-phosphorylating LEAK state (LEAK respiration) with Complex I (CI)-linked substrates (*1PM*) in the MR1 and MRQ1 groups was similar to the control group, but markedly increased after rehabilitation program in both groups of patients (+48% in MR2 vs*.* MR1, *p* = 0.029, and +48% in MRQ2 vs*.* MRQ1, *p* = 0.0013). The increase in LEAK respiration was even higher after additional 12–14 days of CoQ_10_ supplementation (+73% in MRQ3 vs*.* MRQ1, *p* = 0.008) (Figure 3). The respiration at the state of oxidative phosphorylation (OXPHOS) with substrates linked to CI (CI-linked OXPHOS capacity) (*2D*) in the MR1 and MRQ1 groups was considerably reduced vs*.* the control group values (-45.8%, *p* = 0.0008 and -30%, *p* = 0.016). In the MR2 group this parameter slightly increased vs*.* the MR1 group (+12%, ns), in the MRQ2 and MRQ3 groups this parameter associated with adenosine triphosphate (ATP) production significantly increased vs*.* MRQ1 group (+25%, *p* = 0.043 and +50%, *p* = 0.072) (Figure 3). The respiration after addition of cytochrome c *(2D;c)* in the MR1 and MRQ1 group was reduced by 51%, *p* = 0.00004 and 39%, *p* = 0.0005 vs*.* control group values. In the MR2 group this parameter increased by 15% vs*.* MR1 group (ns). In the MRQ2 and MRQ3 groups this parameter significantly increased vs*.* the MRQ1 group (+31%, *p* = 0.012 and +55%, *p* = 0.050). The electron transfer (ET) capacity determined after titration of uncoupler FCCP in optimum concentration *(3U)* was lower in the MR1 and MRQ1 groups vs*.* the control group (-46%, *p* = 0.0003 and -31%, *p* = 0.005). In the MR2 group CI-linked ET capacity increased slightly vs. the MR1 group (+8.8%, ns); in the MRQ2 and MRQ3 groups this parameter increased by 22% (*p* = 0.042) and 42% (*p* = 0.10) vs*.* MRQ1 values (Figure 3). The ET capacity with CI-linked substrates pyruvate plus malate plus glutamate in the MR1 and MRQ1 groups was 40% and 22% lower than in the control group (*p =* 0.001 and *p =* 0.072). This parameter increased slightly in the MR2 group vs*.* the MR1 group (+16%, ns) and markedly in the MRQ2 and MRQ3 groups vs*.* the MRQ1 group (+24%, *p =* 0.029 and +41%, ns). ET capacity with CI&II-linked substrates determined after addition of Complex II (CII)-linked substrate succinate *(5S)* was in the MR1 and MRQ1 groups similar to the control group. This parameter slightly increased in the MRQ2 and MRQ3 groups (+9%, *p =* 0.055 and +15%, ns) in comparison to the MRQ1 group (Figure 3).

**FIGURE 4:**
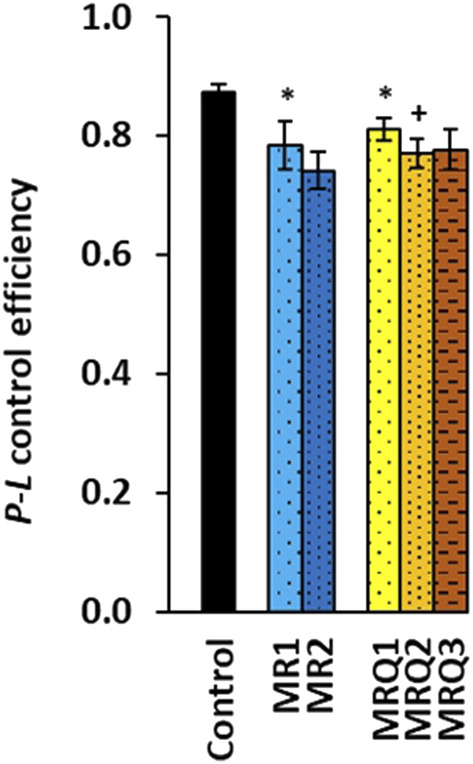
OXPHOS efficiency of platelet mitochondrial respiration of patients with post C-19. *P-L* control efficiency was calculated from parameters of mitochondrial respiration as (2D-1PM)/2D (for parameters and groups refer to Figure 3). *p < 0.05 vs. Control. +*p* < 0.05 vs. the same group at the beginning of the study.

The parameter *P-L* control efficiency calculated from the respiratory capacities as (2D-1PM)/2D was lower in both the MR1and the MRQ1 groups (-10%, *p* = 0.021 and -7%, *p =* 0.038) when compared to the control group and decreased further vs*.* the MRQ1 group in the MRQ2 group (-5%, *p =* 0.041) and in the MRQ3 group (-4%, ns) (Figure 4). This parameter with values from 0 to 1 reflects OXPHOS efficiency with CI-linked substrates—the higher is the value, the higher is the OXPHOS efficiency (Gnaiger 2020). In the MR group the decrease after the rehabilitation program was not statistically significant (Figure 4).

### 3.5 Effect of mountain spa rehabilitation program without/with ubiquinol supplementation on CoQ_10_ concentration and lipid peroxidation in patients with post C-19

**FIGURE 5:**
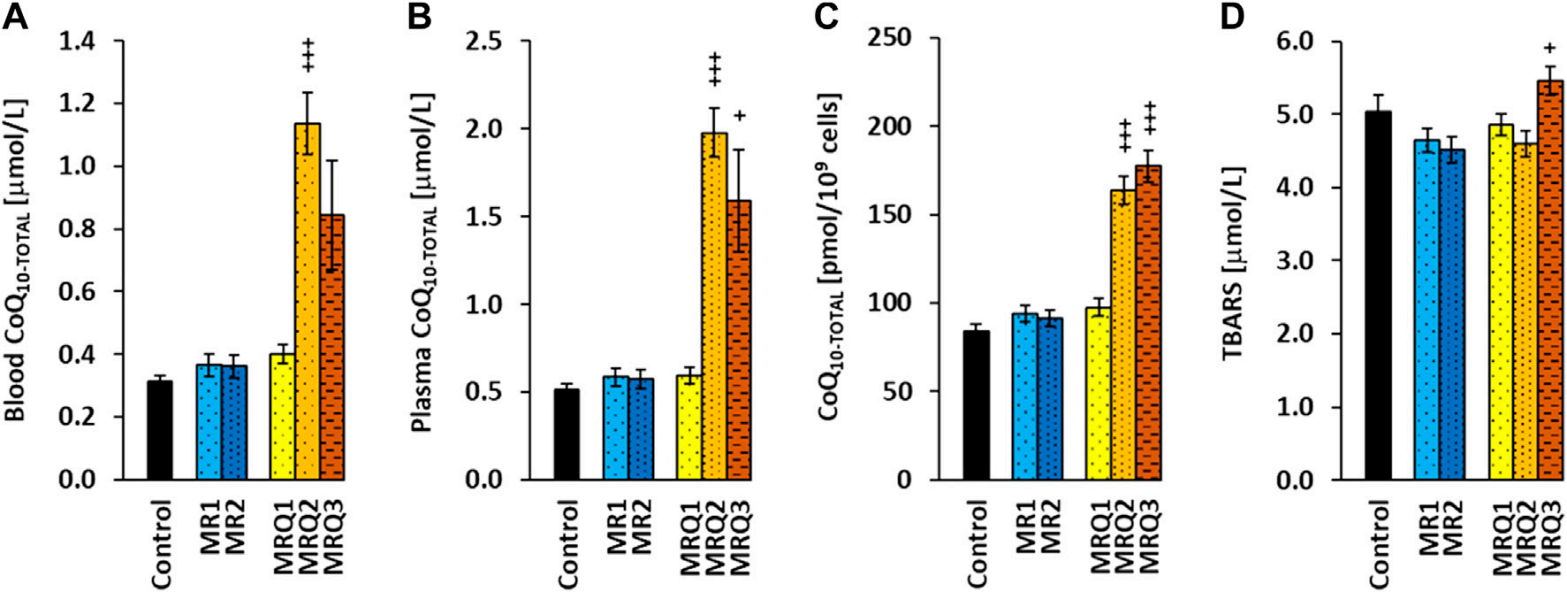
The effect of mountain spa rehabilitation program on CoQ_10_ concentration and lipid peroxidation in patients with post C-19. The concentration of coenzyme Q_10-TOTAL_ in blood **(A)**, plasma **(B)**, platelets **(C)**, and the concentration of TBARS in plasma **(D)** of control subjects and patients with post C-19. The groups names as for Figure 3. +*p* < 0.05, +++*p* < 0.001 vs*.* the same group at the biginning of the study.

The initial concentration of CoQ_10-TOTAL_ (ubiquinol + ubiquinone) in *whole blood* of both groups of patients with post C-19 did not differ from the control group. After supplementation of ubiquinol the concentration of CoQ_10-TOTAL_ in blood markedly increased in the MRQ2 group (+185%, *p =* 0.000003) and in the MRQ3 group (+112%, ns) in comparison with the MRQ1 group (Figure 5A).

Similarly, the concentration of CoQ_10-TOTAL_ in *plasma* of the MR1 and MRQ1 groups did not differ from the control group. In the MRQ2 and MRQ3 groups it was markedly higher (+232%, *p =* 0.0000004 and +167%, *p =* 0.038) than in the MRQ1 group (Figure 5B). The concentration of CoQ_10-TOTAL_ in *platelets* of the MR1 and MRQ1 groups of patients with post C-19 did not differ from the control group and considerably increased in the MRQ2 and MRQ3 groups vs*.* MRQ1 group (+60%, *p =* 0.00006, and +82%, *p =* 0.0009) (Figure 5C). CoQ_10-TOTAL_ concentration in blood, plasma and platelets did not change after rehablilitation program without ubiquinol supplementation. The concentration of TBARS in plasma in all groups of patients was similar to the control group. In the MRQ3 group, TBARS concentration was higher compared to the MRQ1 group (+12%, *p =* 0.031) (Figure 5D).

### 3.6 The correlation between platelet mitochondrial respiration and CoQ_10_ concentration in platelets of patients on mountain spa rehabilitation program with ubiquinol supplementation

**FIGURE 6:**
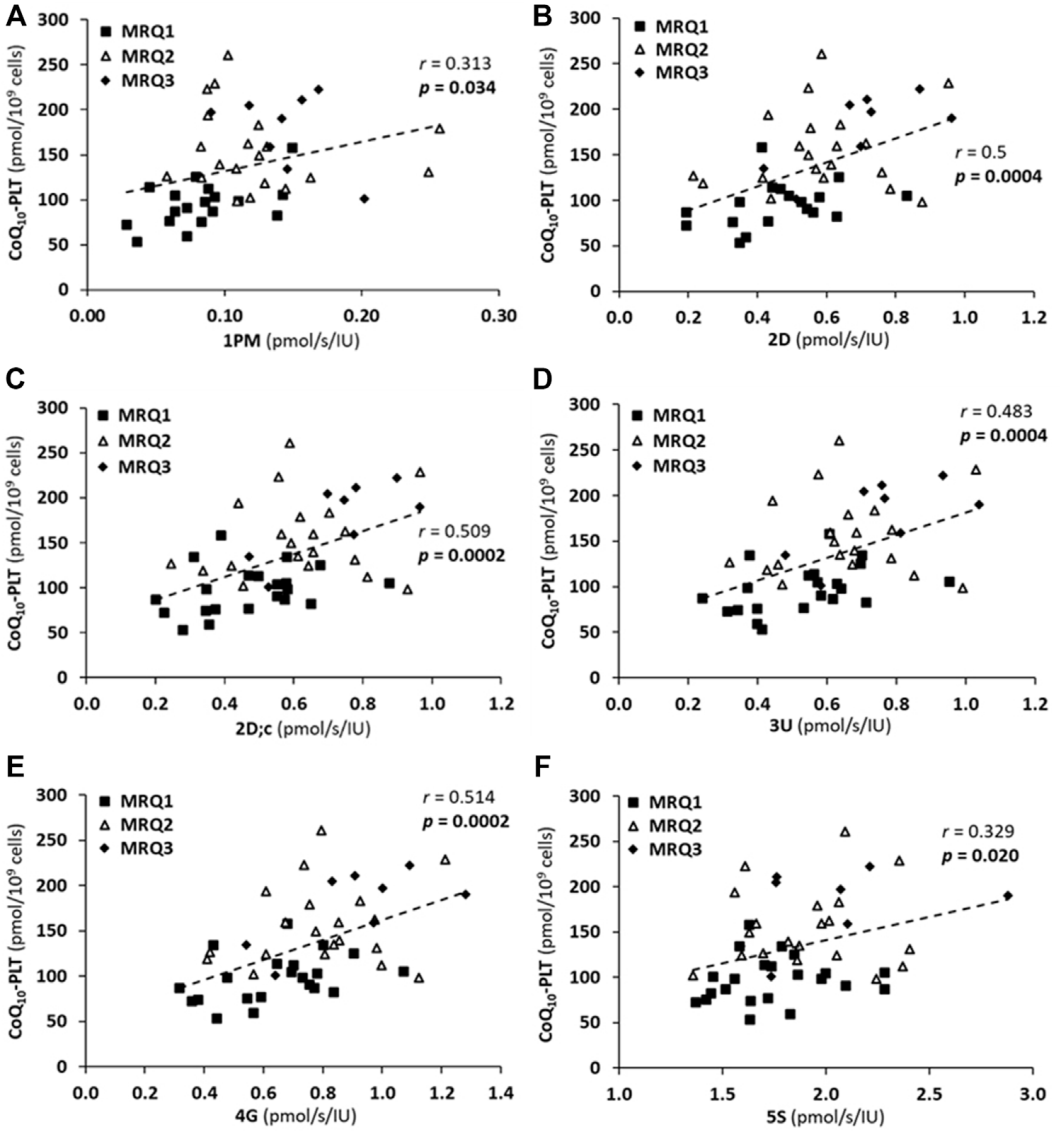
The correlations between CoQ_10-TOTAL_ concentration in platelets (CoQ_10_-PLT) and platelet mitochondrial respiration rates **(A)** 1PM, **(B)** 2D, **(C)** 2D; c, **(D)** 3U, **(E)** 4G, **(F)** 5S of patients with post C-19. The groups names and respiration rates labeling as for Figure 3. *r*—Pearson correlation coefficient; *p*-values show statistical significance of the correlations.

All determined parameters of platelet mitochondrial respiration correlated with CoQ_10_ concentration in platelets when evaluated in all three MRQ groups (MRQ1, MRQ2, MRQ3) together (Figure 6).

There was a significant positive correlation between LEAK (1PM), OXPHOS (2D; 2D; c) and ET (3U; 4G) capacity with CI-linked substrates, as well as ET capacity with CI&II-linked substrates (5S) and CoQ_10_ concentration in platelets in patients of the MRQ group (Figures 6A–F). The Pearson correlation coefficients *r* are valid for the three MRQ groups together, the groups are distinguished by markers for more information. There was not such correlation in the MR group.

### 3.7 The correlation between an increase in platelet CoQ_10_ concentration and an increase in mitochondrial CI-linked OXPHOS and ET capacity in platelets of patients after mountain spa rehabilitation program with ubiquinol supplementation

**FIGURE 7:**
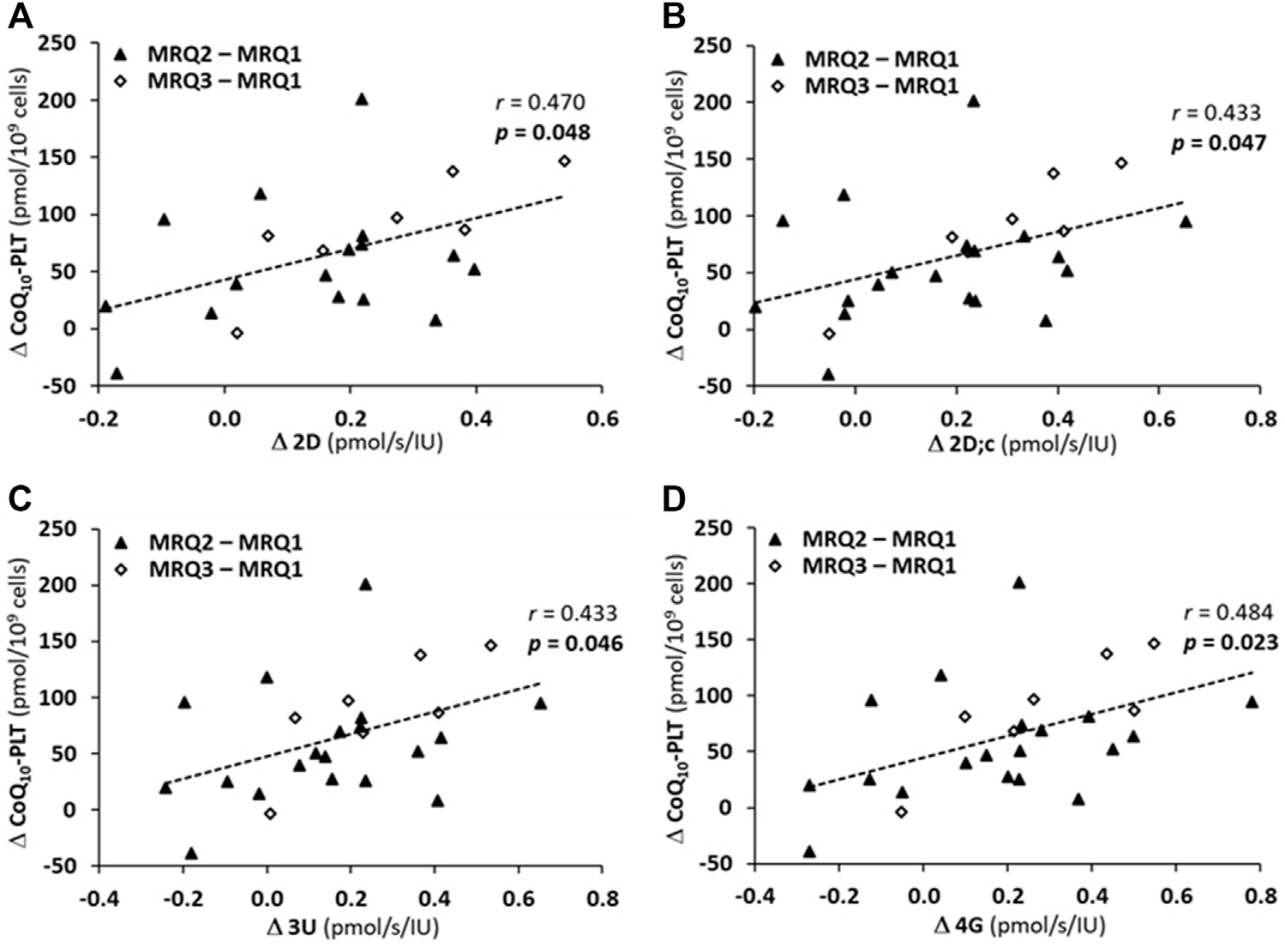
The correlations between an increase of CoQ10 concentration in platelets (ΔCoQ10-PLT) and an increase in parameters of platelet mitochondrial respiration of patients of MRQ group **(A)** Δ2D, **(B)** Δ2D; c, **(C)** Δ3U, **(D)** Δ4G. The markers show the difference values between MRQ2 and MRQ1, and between MRQ3 and MRQ1 time points for each patient from MRQ group. The groups names and respiration rates labeling as for Figure 3. *r*—Pearson correlation coefficient; *p*-values show statistical significance of the correlations.

The correlation analysis showed that the increase in platelet mitochondrial respiration parameters representing OXPHOS (Δ2D, Δ2D; c) and ET (Δ3U, Δ4G) capacity with CI-linked substrates positively correlated with the increase in platelet CoQ_10_ concentration (ΔCoQ_10_-PLT) after the rehabilitation program with CoQ_10_ supplementation (Figure 7).

## 4 Discussion

Three main factors influenced our results: the Mountains (**M**), spa rehabilitation (**R**) and targeted Co**Q**_10_ (ubiquinol) supplementation.

*The Mountains—*the mountain spa rehabilitation has beneficial effect on chronic pulmonary diseases. Sanatorium of Dr. Guhr is located in the High Tatra Mountains, Slovakia at altitude of 1,005 m in the zone of forests with dry air, with a mild favorable solar radiation, and relatively stable daily temperature. Clean air and climatic conditions of High Tatras contribute to successful rehabilitation of patients with chronic pulmonary diseases for many years (Gvozdjáková et al., 2021).

*Spa rehabilitation* - Sanatorium of Dr. Guhr in Tatranská Polianka, Slovakia provides procedures for patients with post C-19, which include respiratory physiotherapy procedures, adequate exercise therapy (Jendrichovsky et al., 2021), mental well-being, and nutrition counseling. Pulmonary rehabilitation programs improve respiratory function, reduce fatigue and dyspnea, and improve exercise endurance and quality of life of patients after COVID-19 (Kołodziej et al., 2021). Beneficial effects of appropriate regular exercise on a large number of chronic diseases is well documented. The regular exercise affects musculoskeletal, immune, hormonal, cardiovascular, and respiratory systems (Posadzki et al., 2020). It has favorable effects on both physical and mental health.

*Ubiquinol—*the reduced form of CoQ_10_. Coenzyme Q_10_ is an essential component of the mitochondrial respiratory chain that transfers electrons from Complex I, Complex II and other electron-transferring proteins to Complex III. CoQ_10_ is a key component in the process of ATP production by oxidative phosphorylation in mitochondria. Ubiquinol is a strong antioxidant protecting cell membranes against oxidative stress. CoQ_10_ is involved in the process of apoptosis, regulates mitochondrial permeability transition pore (Acosta et al., 2016). The health benefit of supplementation with CoQ_10_ has been reported for many diseases including cardiovascular, neurodegenerative, neurological, and psychiatric (Gvozdjáková et al., 2014; Hernández-Camacho et al., 2021; Rauchová, 2021; Sue-Ling et al., 2022). The antiinflammatory, antioxidant properties and supporting effect on mitochondrial function helps in curing infectious diseases (Sifuentes-Franco et al., 2022).

### 4.1 Effect of mountain spa rehabilitation program without/with ubiquinol supplementation on pulmonary function and clinical symptoms of patients with post C-19

After the rehabilitation program in Sanatorium of Dr. Guhr the functional capacity of the lungs significantly improved in both the MR and MRQ groups of patients as seen by increased distance walked during 6MWT and reduced exercise dyspnea assessed by Borg scale (Figure 2). The increase of the walked distance in 6MWT by 70 m and a decrease of exercise dyspnea by 0.5 BS point are considered to reflect clinically significant improvement of lungs function in patients (Casanova et al., 2011). In a study of Liu et al. (2020) on elderly people more than 6 months after overcoming acute COVID-19 the walked distance increased after 6-week pulmonary physiotherapy by 49.6 m. In both the MR and MRQ groups of patients with post C-19 syndrome the improvement of lungs function could be considered clinically significant. The subjective evaluation of the symptoms in the questionnaires showing the reduction of difficulty breathing, and no need of oxygen support after the rehabilitation program corresponds with the results of lungs function tests. The rehabilitation program improved many clinical symptoms such as overall fatigue, muscle and joint pain, heart palpitation, chest pain, headache, and hearing impairment similarly in both groups of patients indicating beneficial effect of rehabilitation procedures, appropriate exercise and diet regimen. Dry cough has improved more markedly in patients supplemented with CoQ_10_, shortness of breath improved only in patients supplemented with CoQ_10_, and overall, higher proportion of clinical symptoms disappeared in the MRQ group. Appropriate regular physical exercise is a non-pharmacological tool for preventing and curing most of the non-communicable chronic diseases (Scheffer and Latini, 2020). Regular practicing of moderate physical exercise activates immune system promoting anti-inflammatory status. The exercise intensity should be sufficient to stimulate biochemical and physiological adaptations to a healthier lifestyle. The potential benefit of individually tailored exercise on the symptoms of post C-19 has recently been reviewed (Jimeno-Almazan et al., 2021). Our data confirm favorable effect of adequate physical activity, an adapted exercise program and diet regimen on lungs function, physical and psychological symptoms of patients after COVID-19.

### 4.2 Effect of the rehabilitation program without/with ubiquinol supplementation on blood count and biochemical parameters of patients with post C-19

Blood glucose and blood lipid parameters were not different from control in groups of patients with post C-19 and were not affected by the rehabilitation program. The patients from the MRQ group had initially higher white blood cells (WBC) count with slightly higher neutrophils (NE) and lymphocytes (LY) count. The LY count was higher compared to the control group also in patients of the MR group. The patients of both groups had higher volume of erythrocytes (MCV) and increased mean corpuscular hemoglobin (MCH) in comparison with the control group. Increased MCV and MCH vs*.* the control group were documented also in non-hospitalized patients 4–7 weeks after diagnosed SARS-CoV-2 virus infection (Sumbalová et al., 2022). MCV, red blood cell distribution width (RDW) and neutrophils count were found to correlate strongly with anti-DNA antibodies present in circulation of COVID-19 patients (Gomes et al., 2021). Cell-free DNA found in high concentration in circulation of COVID-19 patients binds to the surface of endothelial cells, erythrocytes and immune cells and constitutes a target for anti-DNA antibodies triggering the complement-mediated cell lysis (Cheng et al., 2021). The severity of COVID-19 was found strongly associated with the presence of anti-DNA antibodies in patients, and the mortality of hospitalized COVID-19 patients was higher in patients with high RDW (Foy et al., 2020). So, the increased erythrocytes size in patients with post C-19 and increased neutrophil count could indicate increased autoreactivity in these patients. On the other hand, the increase of RBC count, hematocrit and hemoglobin in patients with post C-19 after MRQ may correspond to physiological adaptation to moderate altitude (Basak et al., 2021).

### 4.3 Effect of the rehabilitation program without/with ubiquinol supplementation on platelet mitochondrial function of patients with post C-19

Mitochondria are powerhouses of the cell connecting the metabolism of carbohydrates, amino acids and fatty acids. The virus uses mitochondria for its own replication and the higher energy demand in cells infected by SARS-CoV-2 is supplemented by upregulated aerobic glycolysis (Anand and Tikoo 2013; Zhao et al., 2020). Exact pathobiochemical mechanisms of SARS-Cov-2 virus effect on mitochondrial function are not clear. It is supposed that mitochondria and endogenous production of CoQ_10_ may be the targets of SARS-CoV-2 (Gvozdjáková et al., 2020; Singh et al., 2020). SARS-CoV-2 manipulates mitochondrial energy production indirectly by regulation of ACE2 receptor, and directly by localization of virus open-reading frame protein (ORF)-9b into the host mitochondria causing suppression of innate immunity (Shi et al., 2014; Shi et al., 2018). Many viruses modulate mitochondrial dynamics, metabolism, and mitochondrial bioenergetics, alter intracellular distribution of mitochondria, modulate apoptosis and mitochondrial antiviral immunity (Ripoli et al., 2010; Kaarbo et al., 2011; Vastag et al., 2011; Kalil and Thomas 2019; Tiku et al., 2020). Viral infections induce production of reactive oxygen species (ROS) that may contribute to the alterations in mitochondrial bioenergetics (Ripoli et al., 2010).

Figure 3 shows significantly reduced CI-linked mitochondrial function in platelets of patients with post C-19 of both the MR1 and MRQ1 groups. Platelet mitochondrial CI-linked OXPHOS and ET capacity was in average -48.2% and -42.9% lower in MR1 group, and -34.5% and -26.5% lower in MRQ1 group when compared to healthy controls. After the rehabilitation program CI-linked OXPHOS and ET capacity slightly increased in the MR2 group vs*.* MR1 group (+13.8% and +12.2%, ns) and markedly increased (in average + 28% and +23%) in MRQ2 group vs*.* MRQ1 group. The LEAK respiration with CI-linked substrates increased after the rehabilitation program in both groups by 48%, decreasing slightly the phosphorylation efficiency (-9.5% in MR2 vs*.* MR1, ns, and -5% in MRQ2 vs. MRQ1, *p* = 0.041) (Figure 4). The increase in LEAK respiration after the rehabilitation program indicates higher proton conductance of inner mitochondrial membrane at high protonmotive force. Similar increase in platelet LEAK respiration associated with a decrease in phosphorylation efficiency was reported in ultramarathon runners after the race (Hoppel et al., 2021). Therefore, the observed increase in LEAK respiration may be seen as an adaptation mechanism to exercise preventing increased ROS production by mitochondria.

The results of our study showed that the respiration associated with ATP production *via* oxidative phosphorylation in patients with post C-19 was markedly higher after the rehabilitation program with CoQ_10_ supplementation. The mean increase of CI-linked OXPHOS respiration was +28% in the MRQ2 group and +52% in the MRQ3 group compared to MRQ1 group. The mean increase in CI-linked ET capacity was +23% in the MRQ2 group and +41% in the MRQ3 group when compared to MRQ1 group. These data demonstrate stepwise regeneration of CI-linked mitochondrial function in platelets of patients of the MRQ group. It should be emphasized that all these patients were with depressed mitochondrial function lasting for 3–7 months and this enormous improvement was seen within 1 month of a spa rehabilitation supported by CoQ_10_ supplementation. In platelets of patients of the MR group the mean increase of mitochondrial CI-linked OXPHOS and ET capacity after the rehabilitation program was +13.8% and +12.2% (Gvozdjáková et al., 2022), in both cases representing only half of that achieved during the same time in patients simultaneously supplemented with CoQ_10_.

### 4.4 Effect of mountain spa rehabilitation program without/with ubiquinol supplementation on CoQ_10_ concentration and lipid peroxidation of patients with post C-19

Viral infections induce ROS production and may modulate antioxidant enzymes. Deficit of CoQ_10_ and reduced mitochondrial function can contribute to COVID-19 progression. In non-hospitalized patients 3–6 weeks after COVID-19, reduced endogenous CoQ_10_ levels in whole blood, plasma and platelets were found (Sumbalová et al., 2022). In the present study, no deficit of CoQ_10_ was found in the groups of patients with post C-19 vs*.* the control group (Figure 5). As a consequence of mountain spa rehabilitation supported with CoQ_10_ supplementation, the concentration of CoQ_10-TOTAL_ in whole blood, plasma and platelets significantly increased in the MRQ2 and MRQ3 groups vs*.* the MRQ1 group. In the MR group the concentration of CoQ_10-TOTAL_ in whole blood, plasma and platelets did not change after the rehabilitation program. The high values of CoQ_10_ in blood, plasma and platelets in the MRQ2 and MRQ3 groups show high bioavailability of supplemented ubiquinol which is absorbed by gastrointestinal tract from the small intestine and transported to the liver, into the lymph, blood and tissues of various organs, 58% of CoQ_10_ is incorporated into LDL and 26% into HDL particles (Bhagavan and Chopra., 2006). The slight reduction of CoQ_10_ in blood and plasma of patients in the MRQ3 group compared to the MRQ2 group was associated with an increase of CoQ_10_ concentration in platelets, therefore we assume that longer administration of ubiquinol could lead to its cellular incorporation, and thus reduction in plasma CoQ_10_ level.

The concentration of TBARS in the MR1 and MRQ1 groups was similar to the control group—the parameter of oxidative stress was not elevated as would be expected due to the disease. We suppose that this parameter could be influenced by oxygen therapy and the application of drugs and supplements with antioxidant properties before admission of patients with post C-19 to the Sanatorium. The slight increase of TBARS in MRQ3 group vs*.* MRQ1 group may be related to the lower CoQ_10_ concentration (antioxidant defense) in plasma.

### 4.5 The correlation between platelet mitochondrial respiration and CoQ_10_ concentration in patients of the group on mountain spa rehabilitation program with ubiquinol supplementation

In patients of the MRQ group strong correlation was found between platelet CoQ_10_ concentration and mitochondrial CI-linked OXPHOS and ET capacity (Figures 6B–E). There was also statistically significant correlation between platelet CoQ_10_ concentration and CI-linked LEAK respiration as well as CI&II-linked ET capacity (Figures 6A,F). Further analysis showed also a significant positive correlations between the increase of CoQ_10_ concentration in platelets and the increase of parameters representing OXPHOS and ET capacity with CI-linked substrates (Figures 7A–D) indicating substantial role of supplemented CoQ_10_ in the improvement of mitochondrial CI-linked function after the rehabilitation program. Although the concentration of CoQ_10_ in platelets of patients of the MRQ group was initially similar to that of healthy subjects, the increase of CoQ_10_ concentration significantly contributed to the regeneration of CI-linked OXPHOS and ET capacity in platelets. The systemic increase of cellular CoQ_10_ concentration could positively affect lungs function of these patients.

### 4.6 Sumary

In this study we demonstrated the beneficial effect of a special rehabilitation program on lungs function and clinical symptoms of patients with post C-19. Ubiquinol supplementation during mountain spa rehabilitation program accelerated regeneration of mitochondrial health in patients with post C-19, which was asssociated with better clinical outcome of these patients.

Mountain spa rehabilitation contributed to the regeneration of pulmonary function, the improvement of clinical symptoms and platelet mitochondrial CI-dependent function in patients with post C-19. The supplementation with Coenzyme Q_10_ (ubiquinol) markedly increased concentration of CoQ_10-TOTAL_ (ubiquinol + ubiquinone) in platelets, whole blood and plasma and significantly accelerated regeneration of CI-linked mitochondrial function. The correlation analysis confirmed crucial role of supplemented CoQ_10_ in the regeneration of CI-linked platelet mitochondrial function.

Mountain spa rehabilitation with supplementary CoQ_10_ therapy could be recommended to the patients with post C-19. Platelet mitochondrial function and CoQ_10_ level may be useful markers of mitochondrial health of patients with post COVID-19 syndrome. The result of this pilot study provides another evidence that monitoring of mitochondrial bioenergetics in platelets is a good tool for monitoring of systemic mitochondrial health.

#### 4.6.1 Study limitations

The main limitation of this study is relatively short time of mountain spa rehabilitation (16–18 days), which is sufficient for patients to acquire new movement therapy habits and thus covered by the health insurance company. The total number of patients in the study was limited by the number of patients admitted and treated in the Sanatorium during the period reserved for this study when the necessary instruments from our Laboratory together with 3 workers were temporarily transferred to the Sanatorium (350 km from the Laboratory in Bratislava). The daily limit was a maximum of 6 samples from patients for methodological reasons of working with freshly isolated platelets. Not all patients coming to the Sanatorium with the diagnosis post C-19 wanted participate in the study. The low number of patients with post C-19 in the MRQ3 group (*N* = 8) available for the third time point is another limitation. All these patients were from the Bratislava region and agreed to come to our Laboratory in Bratislava for blood sampling after 1 month of ubiquinol supplementation. Unfortunately, the patients from MR group were not followed up after leaving the Sanatorium.

## Data Availability

The raw data supporting the conclusions of this article will be made available by the authors, without undue reservation.
